# SURGICAL TECHNIQUES TO INCREASE RESECTABILITY IN LIVER METASTASIS

**DOI:** 10.1590/0102-6720202400065e1859

**Published:** 2025-01-20

**Authors:** Orlando Jorge Martins Torres, Guido Torzilli, Marcelo Enne, Rinaldo Gonçalves, Eduardo de Santibanes, Timothy Pawlik, Rene Adam, Olivier Soubrane, Paulo Herman, Ricardo Lemos Cotta-Pereira

**Affiliations:** 1 Hepatopancreatobiliary Surgery and Liver Transplant, Department of Gastrointestinal Surgery – São Luís (MA), Brazil; 2 IRCCS Humanitas Research Hospital, Division of Hepatobiliary and General Surgery – Rozzano, Milan, Italy; 3 Hospital Federal Ipanema, Hospital Samaritano – Rio de Janeiro (RJ), Brazil; 4 Instituto Nacional de Câncer, Abdominopelvic Surgery – Rio de Janeiro (RJ), Brazil; 5 Hospital Italiano de Buenos Aires, Division of Hepato-Pancreato-Biliary Surgery and Liver Transplant Unit, Department of Surgery – Buenos Aires, Argentina; 6 Ohio State University, Wexner Medical Center, Department of Surgery – Columbus (OH), USA; 7 University Paris-Saclay, AP-HP Paul Brousse Hospital, Hepato Biliary Surgery, Cancer and Transplantation Unit – Villejuif, France; 8 Universite Paris Descartes, Institute Mutualiste Montsouris, Oncologic and Metabolic Surgery, Department of Digestive – Paris, France; 9 Universidade de São Paulo, Faculty of Medicine, Department of Gastroenterology – São Paulo (SP), Brazil; 10 D’Or Institute for Research and Education, Digestive Surgery Residency Program – Rio de Janeiro (RJ), Brazil.

**Keywords:** Neoplasm metastasis, Liver, General surgery, Liver transplantation, Metástase neoplásica, Fígado, Cirurgia geral, Transplante hepático

## Abstract

The development of surgical techniques, chemotherapy, biological agents, and multidisciplinary approaches have made patients with unresectable colorectal liver metastases eligible for surgery. Many strategies have been developed to allow patients for surgical resection (percutaneous portal vein embolization, liver venous deprivation, parenchyma-sparing liver surgery, reverse strategy, associating liver partition and portal vein ligation for staged hepatectomy, and liver transplantation), the only form of disease control and curative treatment.

## RESUMO

O desenvolvimento de técnicas cirúrgicas, quimioterapia, agentes biológicos e abordagens multidisciplinares converteram pacientes com metástases hepáticas colorretais irressecáveis (CRLM) para cirurgia elegível. Muitas estratégias foram desenvolvidas para permitir aos pacientes a ressecção cirúrgica (embolização percutânea da veia porta, privação venosa hepática, cirurgia hepática poupadora de parênquima, estratégia reversa, ALPPS, transplante de fígado), a única forma de controle da doença e tratamento curativo.

## INTRODUCTION

The development of surgical techniques, chemotherapy, biological agents, and multidisciplinary approaches have made patients with unresectable colorectal liver metastases (u-CRLM) eligible for surgery. Many strategies have been developed to allow patients for surgical resection, the only form of curative treatment.

### Percutaneous portal vein embolization

Percutaneous portal vein embolization (PVE) is considered the standard of care for inducing hypertrophy in patients requiring a major liver resection and a marginal future remnant liver (FRL). PVE is indicated when the percentage of the total estimated liver volume is ≤20% in a normal liver, ≤30% in a liver with intermediate disease (steatosis, chemotherapy), and ≤40% in a liver with cirrhosis. The technique of interventional radiology is carried out under local anesthesia, and three approaches have been reported: trans-hepatic, trans-splenic, and trans-ileocolic. The degree of hypertrophy after PVE is variable and depends on several factors. Moreover, 20% of patients fail to undergo curative resection, due to either insufficient FRL growth or oncologic progression. The effects of PVE are not observed in patients with severe liver fibrosis and severe acute hepatitis. When the portal pressure after PVE is >30 cmH_2_O, we should attempt another therapy or limit the amount of liver for resection^11,15,22^.

Percutaneous PVE and portal vein ligation (PVL) are comparable in terms of increasing the future liver remnant (FLR) with similar morbidity and mortality according to Pandanaboyana et al.^15^. Both techniques thereby provide an option to the surgeon to be employed in patients with marginal FLR. PVL does not seem to affect the outcome after liver resections^11,15,22^.

### Embolization of segment 4

The embolization of segment 4 (S4) branches leads to increased hypertrophy of the FLR. This procedure is particularly useful for patients with extensive disease and small FLR. In a retrospective cohort study, remnant portal vein embolization (RPVE) + S4 was associated with increased hypertrophy of segments 2 and 3 compared with RPVE alone^8^. The authors recommended that all patients with CRLM in whom extended right hepatectomy is planned and in whom the FRL is small (<20% of the FLR) undergo RPVE + S4. Björnsson et al. observed that the volume of the FLR after PVE including S4 was superior to the volume of the FLR without embolization of S4 (36.7% *versus* 47.9%, p=0.01, p<0.05)^2^. However, portal vein vascularization of S4 varies from 2 to 8 branches between patients and originates particularly from the round ligament and the junction of the left portal vein. Percutaneous PVE of S4 branches is challenging, and its reproducibility has been questioned. Identification of four or more branches of S4 branches could predict the failure of the embolization of S4 branches. Also, there is a risk of accidental thrombosis of the left portal vein when embolizing S4 branches, compromising the FLR^2,8^.

### Liver venous deprivation

Liver venous deprivation (LVD) refers to embolization of the portal vein associated with the hepatic vein (right or right and middle hepatic veins). The LVD technique is well tolerated in patients undergoing right hepatectomy or extended right hepatectomy for CRLM, inducing a higher blood flow in the contralateral liver by also closing the hepatic veins when compared with PVE^7,10^.

A single-center experience from Montpellier observed that LVD seems to be comparable to PVE in terms of oncological results. Data regarding postoperative morbidity and mortality, procedure tolerability, as well as the time between the procedure and surgery after LVD are also promising. However, it is interesting to observe that there are a greater number of postoperative bleeding events in patients who undergo LVD prior to liver resections, although the cases are considered minor bleeding^7,10^.

Kobayashi et al., in a single-center experience, observed that ipsilateral LVD before major hepatectomy is safe and appears to induce a more robust early hypertrophy of the FLR than PVE alone^11^. In a systematic review and network meta-analysis, associating liver partition and portal vein ligation for staged hepatectomy (ALPPS) demonstrated a higher regeneration rate, shorter time to hepatectomy, and a higher resection rate than PVL and PVE^22^. There was no significant difference observed when considering the R0 marge rate. However, there were higher Clavien-Dindo≥3a complication rate and 90-day mortality in ALPPS compared to other treatments. Oncological data should be updated in the coming years, and randomized controlled trials (RCTs) are needed to confirm the benefit of LVD after 5 years of follow-up^7,10^.

### Parenchyma-sparing liver surgery

Modern liver surgeons need to acquire enough level of expertise about the surgical procedures and techniques available to perform a parenchyma-sparing oncological liver resection. Preservation of adequate liver parenchyma is one of the most important issues to prevent postoperative liver failure, which represents the main cause of mortality^1^. Parenchyma-sparing liver resection reduces the rate of postoperative liver dysfunction and increases the possibility of re-resection in the case of recurrence^3^. It is possible to remove the tumor completely with limited and minor resection. Parenchyma-sparing surgery is possible with limited resection for small superficial CRLM (cherry-picking surgery) or minor anatomic resection for lesions located deeply in the parenchyma with intraoperative ultrasound (IOUS) (bisegmentectomy). Furthermore, two major technical procedures have been introduced by Torzilli et al.^17–20^. The first one is to detach CRLM from the major intrahepatic vessels whenever IOUS excludes infiltration. The second includes identification of the communicating vessels among the hepatic veins, which are used to maintain adequate outflow to the liver parenchyma after resection of the main hepatic vein. Parenchyma-sparing surgery has some technical pillars according to Torzilli et al.^17–20^, which include detachment from intrahepatic vascular structures, skeletonization of the Glissonean pedicles, and new anatomic and non-anatomic resections, such as mini-upper transversal hepatectomy, right upper transversal hepatectomy, total upper transversal hepatectomy, mini-mesohepatectomy, liver tunnel, and liver tunnel extended to segment S4^2,3,5,17–20^.

### Reverse strategy

Patients with colorectal cancer and synchronous liver metastases have lower survival rates than those with metachronous colorectal hepatic metastases. There is a controversy regarding the best sequence for resection. There are three strategies in this regard:

- classical approach, which includes colorectal first (after systemic chemotherapy, to obtain better patient selection),
- simultaneous approach, which includes colorectal resection and liver resection during the same procedure, and
- liver first (reverse approach), which includes liver resection first.

The rationale for the liver-first strategy is to control the synchronous metastases, which can optimize a curative hepatic resection and long-term survival. Reverse strategy is indicated for patients with extensive liver metastasis from colorectal cancer who require downstaging therapy and make liver resection possible. Thus, the reverse strategy is an option in the cases of rectal cancer in the early stage and with synchronous liver metastases, or in patients with asymptomatic colorectal cancer, but with extensive liver disease. The patient selection criteria for reverse strategy should be individualized based on approval from the tumor board with a multidisciplinary team^14,21^.

### Associating liver partition and portal vein ligation for staged hepatectomy

**Figure 1:**
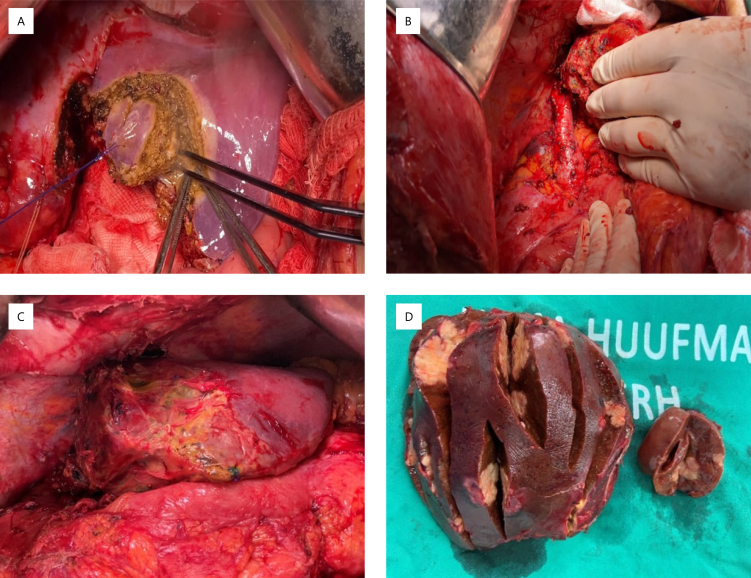
A) right hepatectomy; B) portal vein ligation for staged hepatectomy; C) liver metastasis; D) multiple liver metastases.

Analysis of the international Associating Liver Partition and Portal Vein Ligation for Staged Hepatectomy (ALPPS) registry has observed that CRLM is the best indication for ALPPS in patients with insufficient FLR. Particularly, this population is typically younger, has no underlying liver disease, and has normal portal venous pressure. Multidisciplinary decision-making, neoadjuvant chemotherapy, and adequate patient selection must be employed in patients with borderline resectable CRLM to curative intent resection. Furthermore, a better understanding of tumor biology is paramount to obviate the need for more radical surgery. The best-practice recommendations for ALPPS are as follows: do not undertake ALPPS without a trial of neoadjuvant chemotherapy and avoid ALPPS in patients with a pre-stage I risk score >3. In patients with interstage complications should have stage II delayed, patients with sufficient FLR for primary resection should not undergo ALPPS, consider variants for classical ALPPS (Figure 1A-D), such as partial ALPPS, mini ALPPS, laparoscopic or robotic ALPPS. Patients who have failed sufficient hypertrophy after PVE can benefit from salvage ALPPS. The ALPPS risk score model has been designed to assist surgical decision-making to avoid procedures related to early mortality after ALPPS. The surgeon could postpone stage II surgery or deny ALPPS upfront in cases with a high-risk score that would avoid surgical complications. In some centers, recent experiences indicate continuous improvement of safety, reducing morbidity and mortality comparable to that accepted for major liver surgeries^9,13,16^.

Because of the continually evolving chemotherapy agents and regimens, an ideal number of treatments prior to surgery have been quite heterogeneous. For conversion therapy from unresectable into resectable, the chemotherapy treatment will be dictated by the response to resectable or disease progression. In cases where neoadjuvant chemotherapy is for resectable diseases, some studies suggested that six or fewer cycles might be enough. Furthermore, morbidity has been observed when more than six cycles are utilized^9,13,16^.

The time interval between chemotherapy and liver resection is suggested based on the balance between greater toxicity to the liver if the operation is performed too soon after the last dose of chemotherapy and the risk of disease progression in the case of too long an interval between chemotherapy and surgery. Several heterogeneous studies, particularly retrospective with small groups, consistently suggest an interval between 5 and 8 weeks, depending on the chemotherapy regimen^9,13,16^.

### Liver transplantation

Liver transplantation, an emerging concept of transplant oncology, has been performed for liver tumors in well-selected patients, mainly hepatocellular carcinoma, liver metastases from neuroendocrine tumors, and peri-hilar cholangiocarcinoma. Complete surgical resection remains the treatment of choice for patients with liver metastases, but in a large proportion, it is not possible to obtain a complete R0 resection. In 2006, the Oslo group started the first trial on liver transplantation for patients with colorectal liver metastases (CRLM) (SECA I study). The inclusion criteria were R0 primary colorectal resection, unresectable liver metastases, no extrahepatic disease, at least 6 weeks of chemotherapy, and an Eastern Cooperative Oncology Group (ECOG) performance status 0–1. The overall survival rate at 5 years was 60% with a median survival time of 27 months. Notwithstanding, the disease-free survival rate was 35% in 1 year, and all patients got relapse if observed up to 3 years, mainly in the form of lung metastases which were slow growing and most often resectable. Some factors were identified as related to worse prognosis (the Oslo criteria) and include:

1. Time from primary cancer surgery <2 years;
2. Progressive disease on chemotherapy;
3. Maximum tumor diameter >5.5 cm; and
4. Carcinoembryonic antigen levels >80 μg/l.

Besides Norway, liver transplantation for CRLM has been performed in other countries, including Brazil. Very recently, the Oslo group reported the preliminary results of the SECA II trial, indicating that a 5-year overall survival of about 80% may be obtained if stricter selection criteria for liver transplantation in this patient cohort are used. Nowadays, most liver transplantations reported for u-CRLM utilize deceased donor liver transplant (DDLT) and the Oslo criteria^4,6,12^.

## CONCLUSIONS AND AUTHORS’ COMMENTS

**Moderator:** Please, your considerations about Portal Vein Embolization (PVE)

Paulo Herman: "we actually use glue, especially because of the price of the coils. We must be more liberal indicating PVE. If you are in doubt, do it. I think this is a very important message. In many cases, you'll have 28% or 32% FLR and long-term chemotherapy, in any cases of doubt, do the PVE."

Olivier Soubrane: "I think this is an important message, you should ask your radiologist to embolize very distally especially when you put glue, otherwise you can have some inflammation and a very difficult dissection of the right portal pedicle. And it's even more difficult when you are doing this to laparoscopy."

Rene Adam: "An additional tool is the ratio between the volume of the FLR and the bodyweight (BW) of the patient should exceed 0.5, I mean, the FLR should be 400 CC, for a patient 80 kilogram of BW. The fact that when you are in doubt (about indicate PVE), go ahead, do it, because the security of your hepatectomy will be related to the FLR. It's better to do PVE in a very marginal patient. So, if you doubt do it."

**Moderator:** "And Portal vein ligation (PVL) / alcohol injection: Could you give some comments about that the safety of the procedure?"

Rene Adam: "When we do a two stage hepatectomy we do the PVL. We put a thread, so we ligate and above the thread, we inject alcohol, absolute alcohol in a way to burn the endothelium of the vein. It's much more efficient than a simple PVL."

Eduardo de Santibanes: "If you perform a PVL, when you come in the second stage its really problematic to dissect the hilum. To avoid this, it is better to embolize through the inferior mesenteric vein, in order not to dissect the hepatic hilum."

Paulo Herman: "We prefer the transhepatic approach for PVE. But we have done some cases in which we injected glue instead of alcohol. We had two patients who have had a systemic reaction after the alcohol injection during the procedure. Our routine is percutaneous PVE after the first stage hepatectomy."

Rene Adam: "An additional comment when you inject alcohol, one precaution is to inject around 15 CC for right liver. Please do it very, very smoothly. Because you If you inject with a high pressure at that time, you may induce alcoholic hepatitis."

Timothy Pawlik: "I try not to disrupt the hilum during the first operation, I don't want to dissect the pedicle. Doing a two stage hepatectomy, I would just clear one side and then use PVE percutaneous usually during the same hospital admission."

Olivier Soubrane: "In a two-step conventional procedure, I got the impression that it's better to leave the hilum intact. Few days after clearance (first step), we ask the radiologist to do PVE. For ALPPS there is something that I find useful is to leave some colored tapes quite short, around the PV and the hepatic artery. It's very useful on the second step to find them easier."

**Moderator:** "When you have no growth of your future liver remnants: PVE failure. Do you wait more? Hepatic vein embolization or ALPPS?"

Eduardo de Santibanes: "This is very good question. It can happen. You may have a big volume increase, but the function didn't increase. There is a correlation between volume and function it had been demonstrated in several papers, but not always. So, we must be careful. We must got in function. We are not in a hurry after PVE. We should perform the second step of a two stage or not. So, if the function does not increase, you can continue with chemotherapy and decide when is going to be the best moment to perform the second step."

Rene Adam: "Sometimes we are faced with insufficient volume after one month. So, we may wait up to two months with chemotherapy. Sometimes you do CT scan volumetry, if you see that the volume is increasing, you wait a little bit more, and you obtain what you want. I will wait up to two months at least for saying that the PVE is a failure."

Olivier Soubrane: "When you don't have sufficient hypertrophy, I think it's been important to check on the CT scan that every portal branch has been embolized properly. Sometimes the radiologist has missed a posterior branch. It was a good reason not to get the hypertrophy. Also, I think that if your starting volume is very low you will need serious trigger of regeneration. And usually I don't wait more months, and I'm moving to put a plug in the hepatic vein (liver venous deprivation)."

Timothy Pawlik: "If there was inadequate growth of the of the liver I usually go over it with our interventional radiologists and really make sure that the embolization was adequate. I think Rene Adam point is well taken about waiting for some extra time. But we would also proceed to a hepatic vein plugging (liver venous deprivation) to try to induce additional hypertrophy.

Paulo Herman: "You have to check the quality of your embolization. We are more liberal indicating the deprivation; we don't wait two months. We discuss with a radiologist and if it's a good quality embolization we do the deprivation (hepatic vein embolization)."

**Moderator:** "Is there any case that you go upfront to liver venous deprivation (LVD) as your first strategy? Or you just use it when PVE fails?

Rene Adam: "We still use PVE and if doesn't work, we do venous deprivation. But more and more if the volume is very small, we go upfront to LVD."

**Moderator:** "What do you mean by very small? Do you have a cutoff to indicate LVD?

Rene Adam: "Yes, if you have 15% and less (of FLR) at that time PVE is probably insufficient. And so, LVD is much better."
